# Real-time *GW*-BSE investigations on spin-valley exciton dynamics in monolayer transition metal dichalcogenide

**DOI:** 10.1126/sciadv.abf3759

**Published:** 2021-03-05

**Authors:** Xiang Jiang, Qijing Zheng, Zhenggang Lan, Wissam A. Saidi, Xinguo Ren, Jin Zhao

**Affiliations:** 1 ICQD/Hefei National Laboratory for Physical Sciences at Microscale, CAS Key Laboratory of Strongly-Coupled Quantum Matter Physics, and Department of Physics, University of Science and Technology of China, Hefei, Anhui 230026, China.; 2 SCNU Environmental Research Institute, Guangdong Provincial Key Laboratory of Chemical Pollution and Environmental Safety, and MOE Key Laboratory of Theoretical Chemistry of Environment, South China Normal University, Guangzhou 510006, China.; 3 School of Environment, South China Normal University, University Town, Guangzhou 510006, China.; 4 Department of Mechanical Engineering and Materials Science, University of Pittsburgh, Pittsburgh, PA 15261, USA.; 5 Institute of Physics, Chinese Academy of Sciences, Beijing 100190, China.; 6 Department of Physics and Astronomy, University of Pittsburgh, Pittsburgh, PA 15260, USA.; 7 Synergetic Innovation Center of Quantum Information and Quantum Physics, University of Science and Technology of China, Hefei, Anhui 230026, China.

## Abstract

We develop an ab initio nonadiabatic molecular dynamics (NAMD) method based on *GW* plus real-time Bethe-Salpeter equation (*GW* + rtBSE-NAMD) for the spin-resolved exciton dynamics. From investigations on MoS_2_, we provide a comprehensive picture of spin-valley exciton dynamics where the electron-phonon (e-ph) scattering, spin-orbit interaction (SOI), and electron-hole (e-h) interactions come into play collectively. In particular, we provide a direct evidence that e-h exchange interaction plays a dominant role in the fast valley depolarization within a few picoseconds, which is in excellent agreement with experiments. Moreover, there are bright-to-dark exciton transitions induced by e-ph scattering and SOI. Our study proves that e-h many-body effects are essential to understand the spin-valley exciton dynamics in transition metal dichalcogenides and the newly developed *GW* + rtBSE-NAMD method provides a powerful tool for exciton dynamics in extended systems with time, space, momentum, energy, and spin resolution.

## Teaser

An ab initio nonadiabatic molecular dynamics method based on *GW*-rtBSE is developed to investigate spin-resolved exciton dynamics.

## INTRODUCTION

**Fig. 1:**
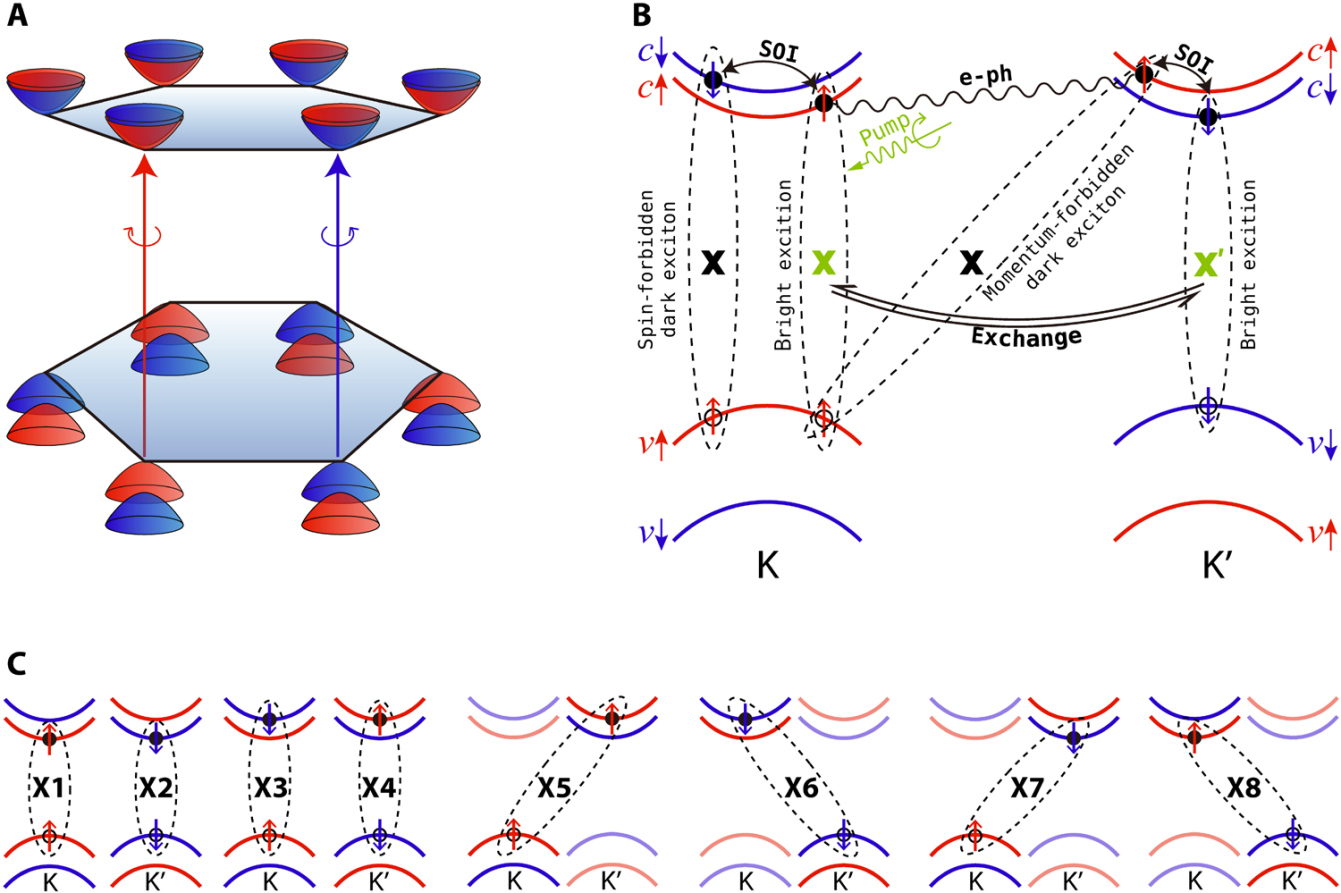
Schematic showing spin-valley dynamics in TMD systems. (**A**) The band structure at the band edges near K and K′. (**B**) The intervalley bright exciton transition and bright-to-dark exciton transition processes are shown. They are induced by e-h exchange, SOI, and e-ph coupling, respectively. (**C**) The e-h pairs involved during the exciton dynamics.

An exciton, a quasiparticle (QP) formed from an electron-hole (e-h) pair bound through Coulomb interactions, represents an optically excited state formed in semiconductors (*1*, *2*). The newly fabricated two-dimensional (2D) materials provide an ideal platform for anomalous excitonic effects to come into play due to the strong quantum confinement and enhanced Coulomb interactions. Thus, exciton dynamics plays an important role in the optoelectronic applications of 2D materials. The most notable example is the 2D transition metal dichalcogenides (TMDs) (*3*–*5*). Because of their hexagonal structure, six valleys are formed at K and K′ points located at the corner of the Brillouin zone as shown in Fig. 1A. The valley states can act as an information carrier, similar to charge or spin. Thus, these valley states are the basic ingredients for valleytronics, which has the potential to offer information processing schemes that are superior to charge- and spin-based semiconductor technologies (*6*–*10*). In monolayer TMDs, the broken inversion symmetry and strong spin-orbit interaction (SOI) lead to spin-valley locking (*6*, *11*), where the spin splitting has opposite signs at the K and K′ valleys. These valley states can be represented by a binary pseudospin and achieve a new ability for exploiting valley polarization. Spin-valley excitons can be optically excited by circularly polarized light at K or K′ valleys, acquiring a valley degree of freedom (*6*–*8*). They play a vital role in the optically driven valleytronic devices based on TMDs.

In TMD monolayers, the bright spin-valley excitons formed by parallel-spin electron and hole in the same valley can be optically excited. The time scale to keep the valley polarization is known as the valley lifetime. After the bright exciton formation, there are different relaxation pathways as indicated in Fig. 1B. First, there can be intervalley bright exciton scattering between K and K′ valleys, which induces valley depolarization. This process is expected to be extremely long because of the spin-valley locking since a large momentum transfer (from K to K′) together with spin flip of both the electron and hole is needed for a valley pseudospin change. However, relatively short valley lifetimes of picosecond time scales have been experimentally observed (*6*, *9*, *12*). Several studies proposed that the e-h exchange interaction activates the intervalley bright exciton scattering (*9*, *13*) and leads to the short valley lifetime. However, the others debate that the electron-phonon (e-ph) scattering is the dominant mechanism (*14*–*16*). Second, a bright exciton can transfer into spin-forbidden dark exciton through the spin flip of the electron or hole, which can be realized through an external magnetic field or internal SOI. It may also transfer to a momentum-forbidden dark exciton with the electron and hole located in different valleys through the scattering with defects or phonons. Compared with bright excitons, dark excitons are found to have appreciably longer valley lifetime and have attracted much attention for potential applications as coherent two-level systems for quantum computing processing and Bose-Einstein condensation (*17*–*22*). However, they are not directly accessible by optical techniques, and how the dark excitons are involved in the valley exciton dynamics is unclear. Furthermore, defects and dielectric properties of the material can also strongly affect the valley exciton dynamics. Long valley lifetimes from tens of nanoseconds to microseconds are found for interlayer excitons (*23*, *24*), and the defect-bound excitons are found to be able to avoid intervalley scattering and achieve microsecond valley lifetime (*25*). Here, different aspects, e.g., e-h interaction, SOI, e-ph scattering, defects, and dielectric properties, come into play in the valley exciton dynamics collectively. To understand such complex valley exciton dynamics quantitatively, real-time ab initio investigations are essential.

In contrast with the rapidly developing time-resolved ultrafast experimental techniques, which have been widely used to study the exciton dynamics in 2D materials, the real-time ab initio methods for exciton dynamics are still in their infancy. The widely used ab initio approach to study the excitons in 2D materials is the *GW* plus Bethe-Salpeter equation (*GW* + BSE) method (*26*–*29*). Although Ismail-Beigi and Louie (*30*, *31*) developed a method to calculate the excited state forces on the basis of *GW* + BSE, making it possible to simulate excited state dynamics, it is mostly used to calculate the “static” properties such as exciton binding energy (*E*_b_) rather than real-time dynamics due to the high computational cost. The ab initio nonadiabatic molecular dynamics (NAMD) based on the time-dependent Kohn-Sham (TDKS) equation combined with Ehrenfest dynamics (*32*–*34*) or surface hopping scheme (TDKS-NAMD) (*35*–*39*) accounts for e-ph coupling and has been successfully implemented to investigate the excited carrier dynamics in condensed matter systems (*37*–*39*). However, the e-h interaction is not included in these approaches. To overcome this disadvantage, recently, the linear response time-dependent density functional theory has been combined with NAMD (LR-TDDFT–NAMD) to investigate the exciton dynamics at TMD heterostructure interface (*40*). E-h interaction is found to play an important role in exciton dynamics. However, LR-TDDFT is proposed to have problems in dealing with excitonic states in extended systems (*28*), and SOI is not included in this approach. In particular, an ab initio method for describing the time- and spin-resolved exciton dynamics that includes e-h, e-ph, and SOI is still missing.

In this work, we develop an ab initio NAMD method based on *GW* plus real-time propagation of BSE (*GW +* rtBSE-NAMD), which is an extension of the TDKS-NAMD with surface hopping scheme (*41*) and classical path approximation (CPA) (*35*). The *GW* method is used to obtain the QP energy levels, and the explicit many-body e-h interaction is included in the real-time propagation of the BSE Hamiltonian. The SOI is included by using the spinor basis sets, and the e-ph coupling is simulated by combining ab initio MD (AIMD) with real-time BSE. To make *GW* + rtBSE-NAMD applicable for the dynamics with hundred thousand time steps for extended systems with hundreds of atoms, we used the rigid dielectric function approximation, in which the dielectric function is obtained by single-point *GW* calculation and assumed not to change during the AIMD simulation. We use *GW +* rtBSE-NAMD to investigate the spin-valley exciton dynamics in monolayer MoS_2_, which is the most intensively studied TMD. It is found that the intervalley bright exciton transition induces fast valley depolarization within a few picoseconds, which is in excellent agreement with previous experiments and model Hamiltonian studies (*9*, *12*, *13*). We provide a direct evidence that e-h exchange interaction plays an essential role in the intervalley bright exciton transitions in TMD systems. In addition, the bright excitons are found to be able to transfer into dark excitons. The e-ph–induced transition to momentum-forbidden dark exciton happens within several tens of femtoseconds, and the transition to spin-forbidden dark exciton is mediated by SOI, which happens within several picoseconds. When the system reaches equilibrium, the system is a superposition of the bright and dark excitons and the population of dark excitons reaches around 67%. The newly developed *GW +* rtBSE-NAMD method provides a powerful tool to investigate the time- and spin-resolved exciton dynamics at the ab initio level.

## RESULTS

### *GW +* rtBSE-NAMD

*GW +* rtBSE-NAMD is analogous to the TDKS-NAMD method with surface hopping by extending the ab initio single-particle KS basis sets to the two-particle e-h pair basis sets applied to BSE. The BSE Hamiltonian is used for the real-time propagation expressed using spinor notation as

$$\langle k\mathrm{cv}|H|k\prime c\prime v\prime \rangle =(E_{kc}^{\text{QP}}-E_{kv}^{\text{QP}})\delta_{kk\prime }\delta_{cc\prime }\delta_{vv\prime }-W_{k\prime c\prime v\prime }^{k\mathrm{cv}}+v_{k\prime c\prime v\prime }^{k\mathrm{cv}}$$ (1)

Here, *v*/*c* represents the index of the hole and electron, while $E_{kc}^{\text{QP}}$ and $E_{kv}^{\text{QP}}$ are the QP energies. $|k\mathrm{cv}\rangle \overset{\cdot }{=}\psi_{kc}(r_{e})\psi_{kv}^{*}(r_{h})$ is the e-h pair basis sets based on spinor formalism. The spinor basis is constructed as $\psi_{kn}(r)=\psi_{↑kn}(r)|↑\rangle +\psi_{↓kn}(r)|↓\rangle \overset{\cdot }{=}\sum_{\sigma =↑,↓}|\sigma kn\rangle ⊗|\sigma \rangle$ through the SOI, in which ψ_↑( ↓ )**k***n*_(**r**) = *e*^*i***k** · **r**^*u*_↑( ↓ )**k***n*_(**r**) is the spin up/down Bloch-like wave functions (see more details in the Supplementary Materials) that can be obtained from the DFT calculations. *W* and *v* represent the screened Coulomb and exchange interaction between the electron and hole, which can be written as

$$\begin{array}{cc} W_{k\prime c\prime v\prime }^{k\mathrm{cv}}= & \frac{1}{\Omega }\sum_{GG\prime }\frac{4{\pi \varepsilon }_{GG\prime }^{-1}(k-k\prime )}{|k-k\prime +G\|k-k\prime +G\prime |}(B_{↑k\prime c\prime }^{↑kc}(G)+B_{↓k\prime c\prime }^{↓kc}(G))\\ & \left(B_{↑k\prime v\prime }^{↑kv*}(G\prime )+B_{↓k\prime v\prime }^{↓kv*}(G\prime )\right) \end{array}$$ (2)

$$\begin{array}{cc} v_{k\prime c\prime v\prime }^{k\mathrm{cv}}= & \frac{1}{\Omega }\sum_{G\neq 0}\frac{4\pi }{{|G|}^{2}}(B_{↑kv}^{↑kc}(G)+B_{↓kv}^{↓kc}(G))\\ & \left(B_{↑k\prime v\prime }^{↑k\prime c\prime *}(G)+B_{↓k\prime v\prime }^{↓k\prime c\prime *}(G)\right) \end{array}$$ (3)

where $\varepsilon_{GG\prime }^{-1}(k-k\prime )$ is the inverse dielectric function that can be obtained from the random phase approximation in the *GW* calculation. **G** is the reciprocal lattice vector, and the Bloch integral *B* defined as $B_{\sigma k\prime n\prime }^{\sigma kn}(G)=\langle u_{\sigma kn}|e^{i\mathrm{Gr}}|u_{\sigma k\prime n\prime }\rangle$ can be calculated from the spinor basis sets.

Using the BSE Hamiltonian of Eq. 1, the time-dependent two-particle Schrödinger equation follows

$$i\hbar \frac{\partial \Phi (r_{e},r_{h},t)}{\partial t}=H(R(t))\Phi (r_{e},r_{h},t)$$ (4)

We followed the TDKS-NAMD method developed by Akimov and Prezhdo (*35*) to use the CPA. On the basis of this approximation, the nuclear motion variable **R**(*t*) in the Hamiltonian is described by the AIMD simulation. Combining with the expansion of the exciton state

$$\Phi (r_{e},r_{h},t)=\sum_{k}\sum_{c}^{\text{elec}}\sum_{v}^{\text{hole}}A_{k\mathrm{cv}}(t)\psi_{kc}(r_{e})\psi_{kv}^{*}(r_{h})$$ (5)

where ψ_**k***c*_(ψ_**k***v*_) are above defined spinor wave functions, we can write the time propagation of the expansion coefficients as

$$i\hbar {\overset{\cdot }{A}}_{k\mathrm{cv}}=\sum_{k\prime }\sum_{c\prime }^{\text{elec}}\sum_{v\prime }^{\text{hole}}(\langle k\mathrm{cv}|H|k\prime c\prime v\prime \rangle -i\hbar \langle k\mathrm{cv}|\frac{\partial }{\partial t}|k\prime c\prime v\prime \rangle )A_{k\prime c\prime v\prime }$$ (6)

The solution of Eq. 6 describes coherent time evolution of excitonic states coupled to the nuclear subsystem, which is expressed by a superposition of the adiabatic basis sets. Such a superposition deviated from the laws of quantum mechanics since the system can only exist in one pure state if measured. To overcome this limitation, a stochastic surface hopping is applied to the system similar to previous studies (*35*, *41*). In this work, we have used the fewest switches surface hopping (FSSH) scheme developed by Tully (*41*). Within the framework of surface hopping, the nonadiabatic coupling elements (NACs) determine the hopping or transition probability from one quantum state to another. In the TDKS-NAMD method, the NAC is calculated as

$$d_{\mathrm{jk}}=\frac{\langle \varphi_{j}^{\text{KS}}|\nabla_{R}H|\varphi_{k}^{\text{KS}}\rangle }{E_{k}^{\text{KS}}-E_{j}^{\text{KS}}}\overset{\cdot }{R}=\langle \varphi_{j}^{\text{KS}}|\frac{\partial }{\partial t}|\varphi_{k}^{\text{KS}}\rangle$$ (7)

in which $\varphi_{j}^{\text{KS}}$ and $\varphi_{k}^{\text{KS}}$ are KS orbitals and $E_{j}^{\text{KS}}$ and $E_{k}^{\text{KS}}$ are KS energies. It can be seen that the e-ph interaction $\langle \varphi_{j}^{\text{KS}}|\nabla_{R}H|\varphi_{k}^{\text{KS}}\rangle$ is captured in the NAC from the KS evolution along with the AIMD (*35*–*38*). In this work, the NAC includes the off-diagonal elements of $\langle k\mathrm{cv}|H|k\prime c\prime v\prime \rangle -i\hbar \langle k\mathrm{cv}|\frac{\partial }{\partial t}|k\prime c\prime v\prime \rangle$ in Eq. 6 where

$$\begin{array}{c} \langle k\mathrm{cv}|\frac{\partial }{\partial t}|k\prime c\prime v\prime \rangle =\{(\langle ↑kc|\frac{\partial }{\partial t}|↑kc\prime \rangle +\langle ↓kc|\frac{\partial }{\partial t}|↓kc\prime \rangle )\delta_{\mathrm{vv}\prime }+\\ {\left(\langle ↑kv|\frac{\partial }{\partial t}|↑kv\prime \rangle +\langle ↓kv|\frac{\partial }{\partial t}|↓kv\prime \rangle \right)}^{*}\delta_{\mathrm{cc}\prime }\}\delta_{\text{kk}\prime } \end{array}$$ (8)

This part contains the e-ph interaction contributed by both the electron and the hole, which is an extension of NAC in TDKS-NAMD. In this approach, the phonon excitation is simulated using AIMD within periodic boundary conditions, suggesting that only phonons at Γ point of the supercell Brillouin zone are included. Therefore, the sampling of phonon momentum depends on the size and shape of the supercell. In this work, we use a 2 × 3 orthogonal unit cell. As shown in fig. S4, there are a total of 12 K points folded into the Brillouin zone. The K, K′, Q, Q′, and Г points, which are important K points for e-ph scattering are all included. Besides the e-ph part, 〈**k***cv*∣*H*∣**k**′*c*′*v*′〉 contains the off-diagonal contribution by *W* and *v* (Eqs. 2 and 3) that is also included in the NAC.

The challenge of the application of this method to large systems and a picosecond time scale is in the *GW* calculation for each time step. To overcome this difficulty, the rigid dielectric function approximation is made in which the dielectric function is assumed not to change during the AIMD simulation. This approximation is reasonable if there is no notable change of the dielectric properties such as insulator to metal during the AIMD simulation. On the basis of this approximation, only a single-point *G*_0_*W*_0_ calculation is needed to obtain the dielectric function. Furthermore, the dielectric function can be calculated using a primitive cell and then transferred to a large supercell as proposed in (*42*). In this work, we perform a single *G*_0_*W*_0_ calculation based on the DFT KS wave function using the Heyd-Scuseria-Ernzerhof (HSE) (*43*) functional (*G*_0_*W*_0_@HSE) using a primitive cell. Here, the energy difference between the Perdew-Burke-Ernzerhof (PBE) (*44*) KS states and *G*_0_*W*_0_@HSE QP energies (Δ*E*_*GW*-PBE_) can be obtained from the single *G*_0_*W*_0_ calculation, and the QP energies in the time-dependent BSE Hamiltonian are obtained using the scissor operator by adding Δ*E*_*GW*-PBE_ to the PBE KS energies. Using scissor operator to get the QP energies was widely applied in previous studies, and it usually works well, especially for the state at the band edges (*27*, *45*, *46*). The validity of the rigid dielectric function approximation is carefully checked, and the results and discussion can be found in the Supplementary Materials.

**Fig. 2:**
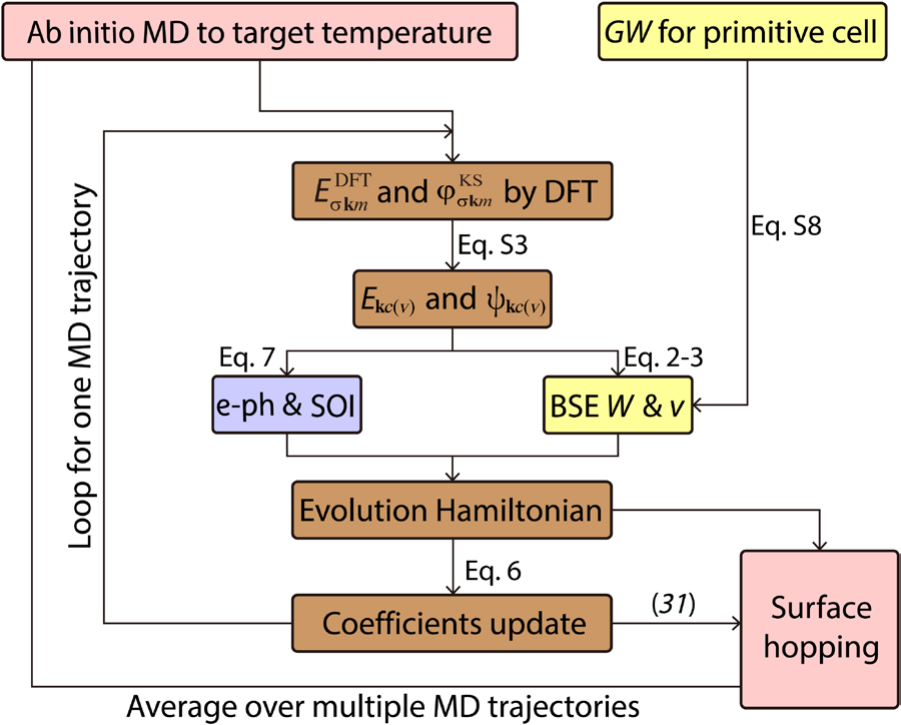
Work chart of current scheme for *GW*-rtBSE-NAMD method. The inner loop works on one AIMD trajectory and the outer loop represents the statistics on samplings of different initial structures.

The *GW* + rtBSE-NAMD workflow can be summarized as follows: (i) Heat the system to a certain temperature and perform an AIMD simulation. (ii) Perform DFT calculations on each structure along the AIMD trajectory to get the time-varying KS orbitals. (iii) Apply the SOI on the KS orbitals to get time-varying spinor–wave function basis sets (eq. S3). (iv) Carry out a *G*_0_*W*_0_ calculation on a single structure to obtain the QP energies and dielectric function. (v) Calculate *W* and *v* on the basis of the time-varying spinor basis sets and dielectric function (Eqs. 2 and 3) and set up the BSE Hamiltonian together with the QP energies. (vi) Solve the time-dependent Schrödinger equation using the BSE Hamiltonian (Eq. 6). (vii) Perform NAMD simulations using a surface hopping scheme based on sampling different initial structures and exciton trajectories to obtain dynamics based on ensemble average. The method is implemented in the Hefei-NAMD code (*36*). A flow chart of the method is shown in Fig. 2.

### QP and exciton binding energies

**Fig. 3:**
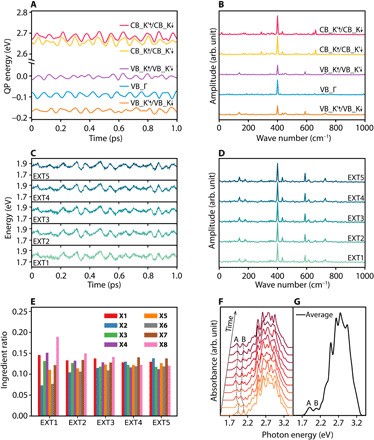
Time-dependent QP and exciton energies. (**A** and **B**) Time evolutions of the QP energies close to CBM/VBM and their Fourier transforms (FTs). (**C** and **D**) Time-dependent energies of the lowest five exciton states (EXT1 to EXT5) and their FTs. (**E**) The averaged ingredients over the MD trajectory for the lowest five exciton states contributed by X1-X8 configurations shown in Fig. 5. (**F**) Snapshots of absorption spectrum along the MD trajectory from *t* = 0 to *t* = 200 fs. The spectrum is plotted in every 20 fs. (**G**) Averaged absorption spectrum along the MD trajectory. The peaks for (A) and (B) excitons are marked.

Before studying the exciton dynamics, it is instructive to check the QP energies and *E*_b_ to verify the validity of the method. Using 2 × 3 orthogonal unit cell, both K and K′ are folded into the Γ point of the supercell. Figure 3A shows the time evolution of QP energies close to the conduction band minimum (CBM) and the valance band maximum (VBM) of MoS_2_. The averaged QP bandgaps (*E_g_*) for MoS_2_ is 2.66 eV, which is in good agreement with previous investigations (*29*, *45*). The phonon excitation simulated by AIMD does not break the time-reversal symmetry, which guarantees the degeneracy of K↑ versus K′↓ and K′↑ versus K↓. Furthermore, the broken inversion symmetry and SOI can induce the splitting at both the CB and the VB. As shown in Fig. 3A, the splitting between CB_K↑/CB_K′↓ and CB_K′↑/CB_K↓ is smaller than 20 meV because of the weak SOI of the CB. By contrast, VB_K↑/VB_K′↓ is higher than VB_K′↑/VB_K↓ by 160 meV because of the strong SOI of the VB. The VB_Γ state is located in the middle of VB_K↑/VB_K′↓ and VB_K′↑/VB_K↓ states. The large spin splitting in the VB ensures that the spin-valley locking is preserved with phonon excitations. This is because the structure distortion in the AIMD simulation does not change the time-reversal symmetry and broken inversion symmetry (*6*, *11*).

The eigenstates obtained from the diagonalization of time-dependent BSE Hamiltonian (labeled as EXT) are the superpositions of different e-h pairs. Because the SOI-induced energy splitting at VB is as large as 160 meV, the VB_K′↑/VB_K↓ and the VB_Γ states barely contribute to the low-energy EXTs. In this case, the two states at the VB (VB_K↑ and VB_K′↓) and the four states at the CB (CB_K↑, CB_K′↓ and CB_K′↑, CB_K↓) combine eight e-h pairs that contribute to the low-energy EXTs. The configurations of the e-h pairs are schematically shown in Fig. 1C. The e-h pairs with parallel spin in the same valley are referred to as bright excitons (K↑K↑ labeled as X1 and K′↓K′↓ labeled as X2). The other six e-h pairs are referred to as dark excitons, in which K↓K↑ and K′↑K′↓ are intravalley spin-forbidden excitons (labeled as X3 and X4), K′↑K↑ and K↓K′↓ are intervalley momentum-forbidden excitons (labeled as X5 and X6), and K′↓ K↑ and K↑K′↓ are the intervalley excitons with both spin and momentum forbidden (labeled as X7 and X8).

In Fig. 3C, we plot the time-dependent energy of the lowest five EXTs (labeled as EXT1 to EXT5). The energy difference between these states is smaller than 0.1 eV. In Fig. 3E, we show the averaged contribution from X1-X8 to the lowest five EXTs over 5 ps. One can see that the bright and dark ingredients are mixed in all these EXTs. In Fig. 3F, we plot the absorption spectrum snapshots from *t* = 0 to *t* = 200 fs with the averaged absorption spectrum over the MD trajectory shown in Fig. 3G. Here, the averaged energy for the lowest A exciton peak for MoS_2_ is 1.91 eV. The corresponding *E*_b_ can be obtained by subtracting energy of A exciton from *E*_g_. The averaged binding energies for A exciton in MoS_2_ are around 0.75 eV during the 5-ps AIMD simulation. These results are in good accordance with previous theoretical and experimental results (*29*, *45*).

### Exciton-phonon interaction

The fluctuations of the QP and EXT energies indicate e-ph coupling contributed by the electron or hole (Fig. 3, A and B) and the exciton (Fig. 3, C and D), respectively. The amplitudes of the energy fluctuations characterize the strength of the e-ph interaction, while the Fourier transform (FT) spectra reveal the dominating phonon mode frequencies contributing to the e-ph coupling. As shown in Fig. 3B, the major phonon peak coupled with the electron and hole is the optical A_1_ mode at 400 cm^−1^. The acoustic phonon modes around 200 cm^−1^ also contribute to e-ph coupling (*47*). These assignments are in agreement with previous AIMD investigation (*37*). In addition, there are additional minor peaks around 600 and 700 cm^−1^, which is a combination of the optical A_1_ mode and the acoustic mode.

The exciton-phonon interaction can be understood from the FT spectra of the lowest five EXTs shown in Fig. 3D. The major phonon peaks are still located at 400 and 200 cm^−1^. The combinatorial phonon peaks at 600 and 700 cm^−1^ are stronger, which may account for the e-h interaction in an exciton. The multiplication of the electron and hole wave functions in the calculations of the Coulomb and exchange interaction (*W* and *v*) as shown in Eqs. 2 and 3 can enhance these combinatorial modes.

### Valley exciton dynamics

**Fig. 4:**
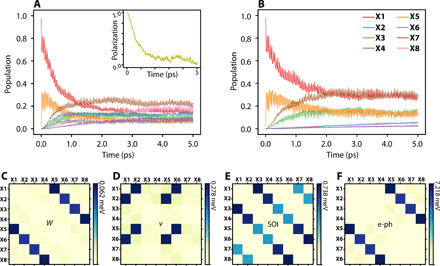
Dynamics results and nonadiabatic couplings. (**A** and **B**) Time evolution of the population on X1 to X8 (A) with and (B) without the e-h interaction *W* and *v* in the NAMD simulation. The time-dependent valley polarization is inserted in (A). (**C** to **F**) Averaged NACs contributed by *W*, *v*, SOI, and e-ph, respectively.

In MoS_2_, the bright exciton X1 can be excited by circularly polarized light, and it is set as the initial excitation in our investigation. The time-dependent population on different exciton states is shown in Fig. 4A. The fluctuations of these curves suggest that the exciton decay couples with phonon excitations, and through FT spectra, we can extract the related phonon information (see fig. S3). It is found that the combinatorial phonon peak is around 600 cm^−1^, which is the combination of the optical A_1_ and acoustic modes, playing a dominating role in coupling with the exciton decay curve. After the excitation, the population of X1 decays from 98 to 68% within 30 fs, which is half of the period of the phonon peak at 600 cm^−1^. Simultaneously, the population of intervalley momentum-forbidden exciton X5 increases from 0 to 30%. Such an ultrafast process corresponds to the intervalley e-ph scattering of the electrons between CB_K↑ and CB_K′↑. As discussed above, the SOI-induced energy splitting between CB_K↑ and CB_K′↑ is small, and therefore, such intervalley e-ph scattering is able to occur. In agreement with our results, such an ultrafast intervalley electron scattering with phonon in TMD systems was observed by two different groups using time- and angle-resolved photoemission spectroscopy (*48*, *49*), in which the e-ph scattering time scale was reported to be smaller than 60 fs. After that, the populations of both X1 and X5 start to decay in a slower manner and, at the same time, the populations on X7, X8, and X2 begin to increase. After around 4 ps, the system reaches the equilibrium. The population of X7 and X8, which are the two intervalley excitons with both spin and momentum forbidden, has the highest population, which are 22 and 18%, respectively. It is because these two exciton states have the lowest energy due to the SOI-induced energy splitting at the CB and the lack of exchange energy. The two bright excitons X1 and X2 host 18 and 15% population, respectively. The remaining population distributes over the other four dark excitons.

The intervalley bright exciton transition from X1 to X2 corresponds to the depolarization process observed by experiments (*6*, *9*, *12*). The time-dependent polarization can be calculated as

$$\eta (t)=\frac{n_{X1}(t)-n_{X2}(t)}{n_{X1}(t)+n_{X2}(t)}$$ (9)

where *n*_X1_(*t*) and *n*_X2_(*t*) are the time-dependent populations of X1 and X2, respectively. As shown in the inset of Fig. 4A, the polarization of the bright valley exciton decays to almost zero within 5 ps. Experiments based on ultrafast transient absorption spectroscopy and time-resolved photoluminescence reported valley depolarization time scales in MoS_2_ to be below 10 ps (*9*) and around 4 ps (*12*), which are in good agreement with our results.

### Exciton transition mechanism

**Fig. 5:**
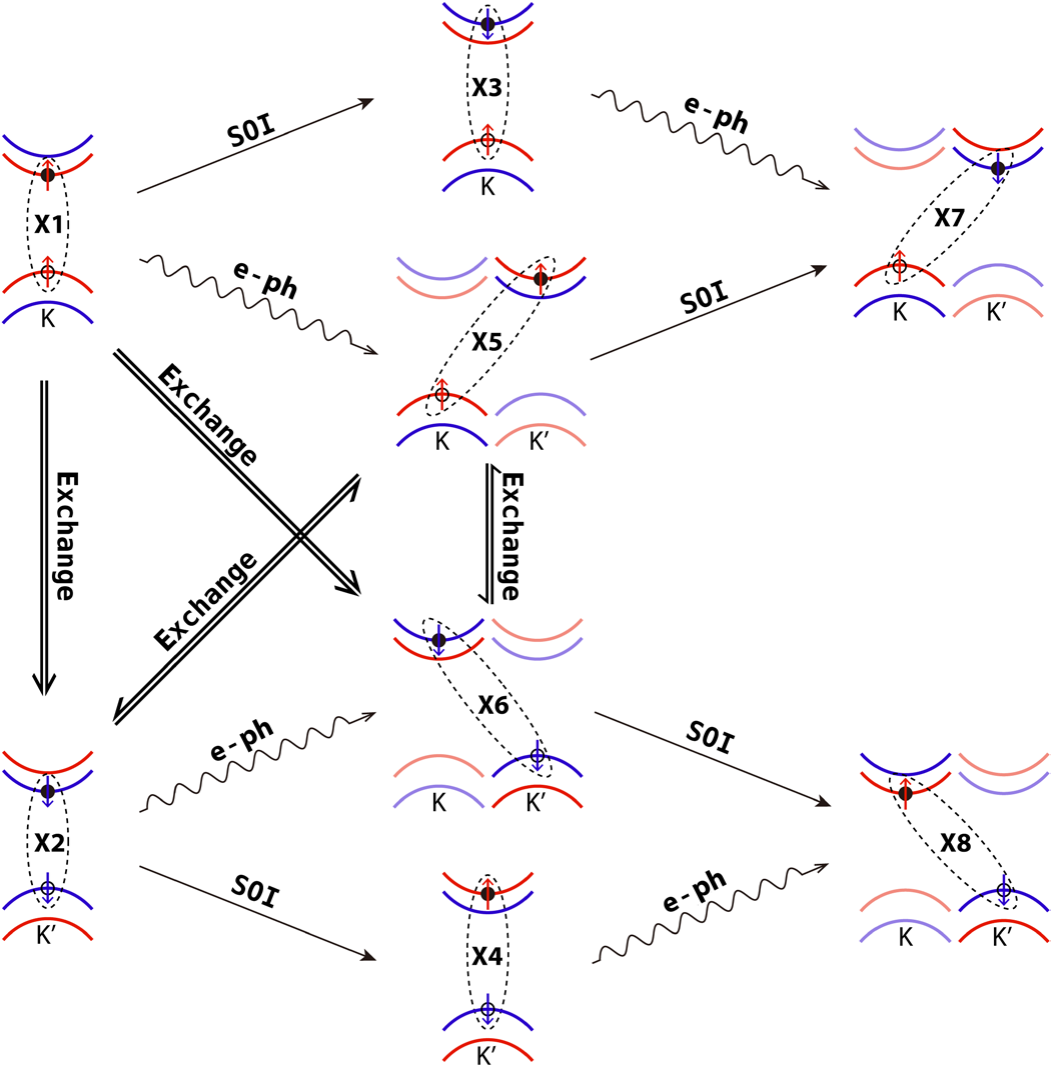
Schematic map of the exciton dynamics channels and the correlated mechanisms. The excitons can be divided into two groups with different hole status: X1, X3, X5, and X7 with K↑ hole and X2, X4, X6, and X8 with K′↓ hole. The SOI and e-ph contribute to the exciton transition within one group, and the exchange interaction contributes to the exciton transition between different groups.

The exciton transition dynamics from X1 to other excitons are schematically shown in Fig. 5. The eight excitons can be divided into two groups. X1, X3, X5, and X7 belong to the first group, in which the hole is located in K valley. X2, X4, X6, and X8 belong to the second group, in which the hole is located in K′ valley. As discussed above, the X1-X5 transition first occurs within 30 fs, which is due to intervalley e-ph coupling between K and K′ valley at CB. In this process, the phonon compensates the change of the exciton momentum, but there is no spin flip. The SOI is responsible for the coupling between the spin-parallel (bright) and spin-antiparallel (dark) exciton states. After X5 is populated, the X5-X7 transition happens because of the SOI. In addition, there is another minor transition route as X1-X3-X7, which is mediated first by SOI and then by e-ph scattering. If only SOI and e-ph scattering exist, then the exciton transition can only happen among the first exciton group, where the hole always locates in K valley.

The many-body e-h exchange interaction *v* determines the coupling between the spin-parallel *e*↑*h*↑ and *e*↓*h*↓ excitons. Because of the exchange interaction, X1-X2, X1-X6, X5-X2, and X5-X6 can happen, and the bright exciton X2 in K′ valley can be populated. After X2 is populated, X2-X4-X8 and X2-X6-X8 can happen because of SOI and e-ph scattering. Then, the excitons in the second group can be populated. It can be seen that the exchange interaction determines the coupling between the first and second groups. The valley depolarization that corresponds to the transition of X1-X2 is induced by the exchange interaction.

In NAMD simulation, the NACs, which are the off-diagonal elements of the time-dependent Hamiltonian, determine the transition rate between different states. As we discussed above, in the *GW*-rtBSE-NAMD simulation, the e-ph, SOI, and the e-h interaction including Coulomb interaction *W* and exchange interaction *v* all contribute to the NAC elements. In Fig. 4 (C to F), we plot the partial contribution of NAC by *W*, *v*, SOI, and e-ph, respectively. One can see that e-ph coupling has the largest value, which is one order of magnitude larger than SOI and *v*. It explains the ultrafast e-ph scattering within several tens of femtoseconds between X1 and X5. The SOI and *v* have the same magnitude. That is why the X1-X2 and X5-X7 process has a similar time scale of several picoseconds. The contribution of *W* to the NACs is the smallest; it is one order of magnitude smaller than SOI and *v*, and it has the same trend as the e-ph coupling. Note that *W* modifies the diagonal elements of BSE Hamiltonian notably, which is the major contribution of the exciton binding energy, but it barely influences off-diagonal elements, which corresponds to the coupling between different excitons.

## DISCUSSION

Our results clearly show that the e-ph, SOI, and e-h exchange interactions collectively affect the valley dynamics in TMD. In particular, the e-h exchange interaction, which belongs to many-body effects, is essential to understand the valley depolarization in TMDs. This is distinctly different from the dynamics of a single particle. To further understand this point, we perform another NAMD simulation by setting the values of e-h interaction *W* and *v* to be zero. The results are shown in Fig. 4B. As expected, after excitation, X1 can only transfer to X5, X7, and X3 by e-ph and SOI. The excited hole is kept in K valley. That is why when the e-h exchange interaction is suppressed, e.g., for interlayer excitons, dark excitons, defect-bound, and resident carriers, they usually have much longer valley lifetime (*23*–*25*, *50*–*52*). The key role of e-h exchange interaction in valley exciton dynamics is also in accordance with the recent work by Guo *et al.* (*53*), in which the e-h exchange is shown to drive the mixing of the *c*↑*v*↑ and *c*↓*v*↓ excitons in the same valley. Our work provides direct evidence for the e-h exchange–induced valley depolarization mechanism at the ab initio level.

Moreover, our work provides a clear description of how the spin-forbidden and momentum-forbidden dark excitons formed in MoS_2_. After equilibrium, the bright excitons only keep around 33% population, while the dark excitons hold 67% of the total population. The dark excitons have long lifetime and are possible to be switched and modulated, offering new strategies for quantum optoelectronics (*21*, *54*).

*GW* + BSE calculations were proposed to be prohibitively expensive to combine with NAMD simulations (*40*). In this work, we introduce rigid dielectric function approximation to overcome this difficulty and realize the *GW* + rtBSE-NAMD simulation for TMD systems. It is assumed that the dielectric environment does not change substantially during AIMD simulation. In this work, we have carefully tested the validity of this approximation, as shown in fig. S2. We propose that this approximation works well for most solids below their phase transition temperatures. For liquids and molecules, since the structure distortion is expected to be much more distinct than solids during the AIMD simulation, this approximation may be invalid.

The newly developed *GW* + rtBSE-NAMD method provides a powerful tool to investigate the time- and spin-resolved exciton dynamics, including the e-h, e-ph, and SOI. Our approach is different from the previous nonequilibrium Green’s function and the TDBSE methods (*55*–*57*), in which the time propagation is on the Green’s function *G* rather than on the BSE Hamiltonian. Comparing with the ab initio TDKS-NAMD, the many-body e-h interaction is explicitly considered here using time-dependent BSE. Moreover, the spin degrees of freedom are included by taking account of SOI. In TDKS-NAMD, the e-ph coupling is included by coupling TDKS with the AIMD through the NAC calculation (*35*–*37*). Here, the e-ph is treated in a similar way, yet the contributions of both electron and hole are included on equal footing by explicitly accounting for exciton-phonon interaction. Comparing with LR-TDDFT–NAMD developed by Liu *et al.* (*40*), LR-TDDFT–NAMD has the advantage in computational cost yet the *GW* + rtBSE-NAMD is believed to be able to treat the many-body e-h interaction more accurately in extended systems. For liquids and molecular systems, if the rigid dielectric function approximation does not work, then LR-TDDFT–NAMD is a good choice to include the e-h interaction.

In contrast with previous investigations on exciton dynamics using parameter-dependent model Hamiltonian (*13*, *58*), the ab initio *GW*-rtBSE-NAMD sheds critical insights at the atomistic level. It is able to provide a quantitative and comprehensive description of various interactions (e-h Coulomb and exchange, SOI, and e-ph) in different materials. We propose *GW*-rtBSE-NAMD to be an indispensable tool in exploring the large and increasing number of 2D materials, including complex structures such as defects, impurity doping, heterostructures, molecular adsorption, and so on, and their potential connections with valleytronic devices. Furthermore, the application of this method is not limited to 2D materials. We expect that it will pave a new way to study exciton dynamics in solid materials at the ab initio level.

## MATERIALS AND METHODS

The *G*_0_*W*_0_@HSE calculation, AIMD simulation, and DFT level calculations to get the KS orbitals are carried out using the Vienna Ab initio Simulation Package (*59*) with projector-augmented wave pseudopotentials (*60*) and 400-eV energy cutoff. The *G*_0_*W*_0_@HSE calculation is performed on a primitive cell with 24 × 24 × 1 Г-centered K-point grid and 256 bands. For the AIMD simulation, to get the nuclear trajectory and DFT calculations to obtain the KS wave functions, we use a 2 × 3 orthogonal unit cell with 6 × 8 × 1 k-point grid based on PBE functional. After the geometry optimization, we use velocity rescaling to bring the temperature of the system to 100 K. A 5-ps microcanonical AIMD trajectory is then generated from which the time-dependent KS wave functions can be obtained. The time intervals are 1.0 and 10^−3^ fs for the ions in AIMD and excitons in real-time BSE, respectively. After the real-time propagation of BSE, the surface hopping is applied on the basis of FSSH scheme using, on average, 100 initial structures and 2 × 10^4^ trajectories for each structure. More details can be found in the Supplementary Materials.

## SUPPLEMENTARY MATERIALS

Supplementary material for this article is available at http://advances.sciencemag.org/cgi/content/full/7/10/eabf3759/DC1
